# Development of Dual-Activity Vectors by Co-Envelopment of Adenovirus and SiRNA in Artificial Lipid Bilayers

**DOI:** 10.1371/journal.pone.0114985

**Published:** 2014-12-12

**Authors:** Açelya Yilmazer, Bowen Tian, Kostas Kostarelos

**Affiliations:** 1 Nanomedicine Laboratory, Faculty of Life Sciences, University College London, London, United Kingdom; 2 School of Pharmacy, University of Nottingham, Nottingham, United Kingdom; 3 Faculty of Medical & Human Sciences and National Graphene Institute, University of Manchester, Manchester, United Kingdom; University Claude Bernard Lyon 1, France

## Abstract

Gene therapy with human adenovirus type 5 (Ad5) has been extensively explored for the treatment of diseases resistant to traditional therapies. Intravenous administration leads to rapid clearance from blood circulation and high liver accumulation, which restrict the use of Ad-based vectors in clinical gene therapy protocols that involve systemic administration. We have previously proposed that such limitations can be improved by engineering artificial lipid envelopes around Ad and designed a variety of artificial lipid bilayer envelopes around the viral capsid. In this study, we sought to explore further opportunities that the artificially enveloped virus constructs could offer, by designing a previously unreported gene therapy vector by simultaneous envelopment of Ad and siRNA within the same lipid bilayer. Such a dual-activity vector can offer efficacious therapy for different genetic disorders where both turning on and switching off genes would be needed. Dynamic light scattering, transmission electron microscopy and atomic force microscopy were used to characterize these vectors. Agarose gel electrophoresis, Ribo green and dot blot assays showed that siRNA and Ad virions can be enveloped together within lipid bilayers at high envelopment efficiency. Cellular uptake and *in vitro* transfection experiments were carried out to show the feasibility of combining siRNA-mediated gene silencing with viral gene transfer using these newly designed dual-activity vectors.

## Introduction

Gene therapy is currently being evaluated for a wide range of genetic disorders including cancer, cystic fibrosis (CF), Parkinson's disease (PD), sickle cell anemia, amyotrophic lateral sclerosis (ALS), etc. Over the last decade, the gene therapy community has recognized that a general vector for all genetic disorders will be difficult to discover, hence the gene transfer agent has to be carefully chosen depending on a number of aspects such as: genetic condition of the disorder, targeted cell type, required number of treatments (one versus repeat administration), and size and nature (secreted versus cellular product) of the gene to be delivered. In some cases, a rigorous treatment strategy might be also needed which involves both the overexpression of a functional transgene (gene delivery) in combination with the simultaneous silencing of the diseased gene (siRNA/shRNA delivery) whose overexpression may be involved in the pathogenesis.

Oncolytic adenoviruses, which are one of the most widely studied vector in cancer treatment, replicate selectively in cancer cells and destroy the cells through cell lysis. An interesting approach to increase the antitumour activity of oncolytic viruses has been achieved through the use of RNAi. This double targeting approach has recently become more popular and involved the combined effect of oncolysis by adenoviruses and siRNA silencing. The existence of a shRNA expression cassette (for example, against the mutated K-rasV12 oncogene) within a viral genome has shown to increase the inhibitory effect on tumour growth. According to Zhang et al. siRNA delivery by an oncolytic virus has many appealing features in terms of vector development and anti-tumour activity [1]. Zhang et al. showed that co-treatment of A549 cells with ONYX-411 and siKRas, led to more than 90% of cell death. Furthermore, the combined activities of siKRas and viral oncolysis produced significant anti-tumour activity *in vivo* [1]. Another study showed that co-treatment with siRNA increased DNA replication of oncolytic Ad and, thus enhanced the spread of virus in the tumour environment [2]. This technology has also been extended to other genes which are upregulated in cancer [3]–[5]. Overall, the results of these studies indicate that the use of oncolytic Ads to deliver tumour-targeted shRNA offers multiple potential benefits.

We have previously reported that a variety of artificial lipid envelopes can be engineered around adenoviral capsid in order to alter the vector characteristics [6]–[8]. In this study, we aimed to develop a novel dual-activity vector by envelopment of Ad and siRNA within a single lipid bilayer. We hypothesised that these vectors can offer efficacious therapy capabilities for complex genetic disorders where an efficient therapy would require both ‘turning on’ simultaneously with ‘switching off’ of specific genes. This report offers proof of concept of a dual-activity vector using the β-Gal and luciferase reporter gene systems.

## Results

**Figure 1:**
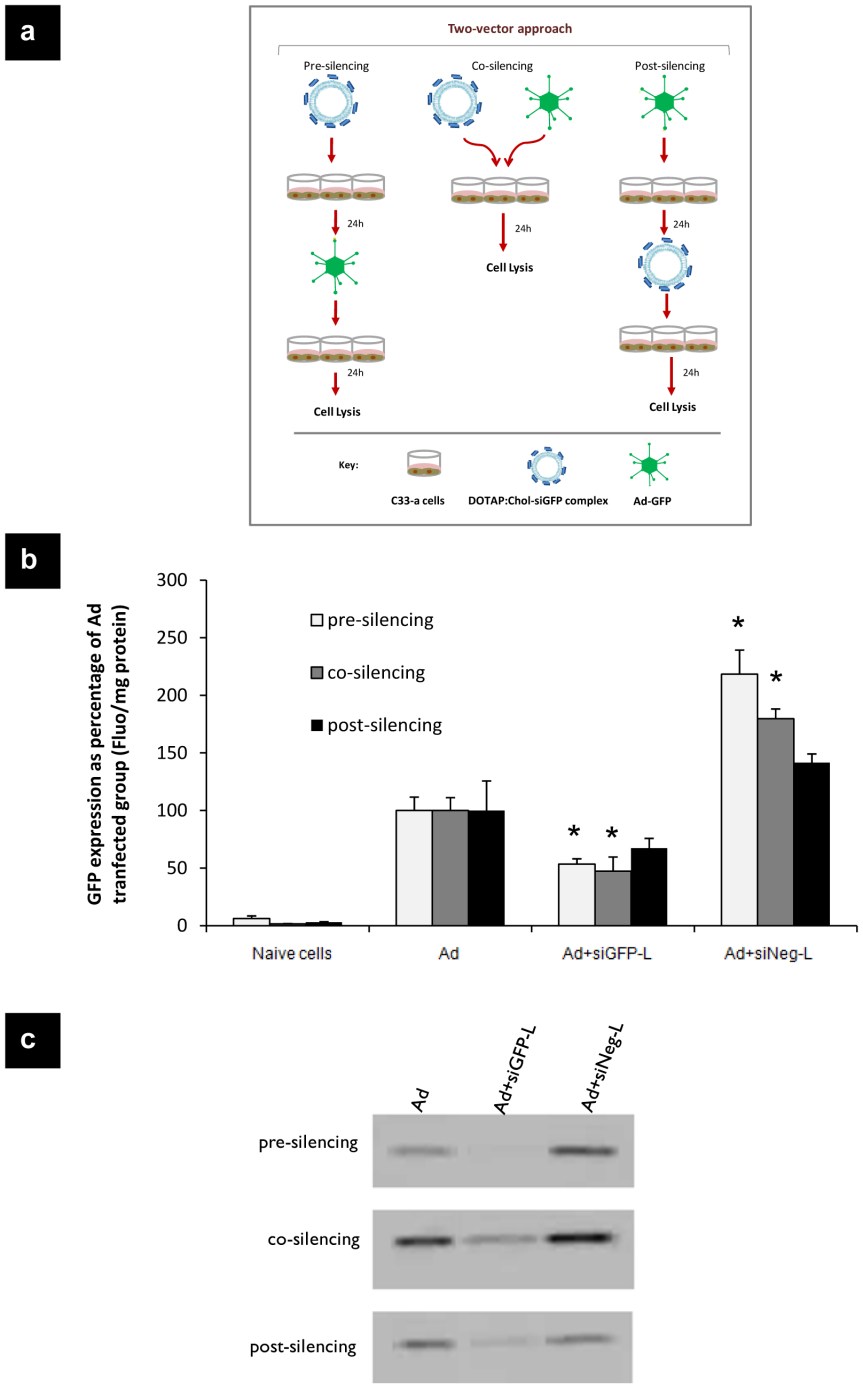
“on and off” gene expression *in vitro* with Ad-GFP transfection and siGFP silencing. (a) Schematic representation of two-vector treatment protocols. For the two-vector approach *in vitro* cell transfections, there are three methods: pre-, co- and post- silencing. In pre-silencing, cells are treated firstly with siRNAs complexed to DOTAP:Chol liposomes and after 24 h Ads are added to the cells. In co-silencing protocol, cells are co-incubated with liposome-siRNA complexes and Ad. For post –silencing, cells are treated with liposome-siRNA complexes 24 h after Ad transfection. Cells are lysed after the last treatment in each silencing group and analyzed for gene expression. (b) C33-a cells were subjected to Ad-CMV-GFP (108 particles/mL) transfection and DOTAP:Chol liposomes complexed to siRNA (L-siGFP or L-siNeg, at 60 nM concentration) treatments at different time points as described. The cells were lysed 24 h after the last treatment and fluorescence per mg protein was measured to plot GFP expression as percentage of Ad transfected group. (c) Cell lysates were also run on SDS-PAGE electrophoresis and blotted to reveal the differences in protein levels in each treatment group. * p<0.05, ** p<0.01, *** p<0.001 versus Ad.

First, the feasibility of gene delivery by Ad with the subsequent gene silencing by siRNA independently were studied. We tested three different gene silencing treatment protocols: pre-, co- and post- silencing (Fig. 1) using a viral vector (Ad-GFP) to transduce cells and a non-viral vector (DOTAP:Chol-siRNA liposome complexes) for gene silencing. Fluorescence per mg protein as percentage of the Ad transfected group indicated that ‘pre-silencing’ and ‘co-silencing’ treatment groups both showed significantly lower GFP expression compared to the Ad control group (p<0.001 and p<0.01 versus Ad, respectively). Gene knockdown efficiency by the’ post-silencing’ protocol was lower than ‘pre- or co-silencing’ and there was no statistical difference compared to Ad (p>0.05). Treatment with siNeg did not result in gene silencing but showed either the same levels of GFP expression compared to Ad alone or much higher levels of GFP expression (Fig. 1b).

In addition to the fluorescence intensity data, western blotting was performed to reveal any differences in protein level (Fig. 1c). A comparison between bands corresponding to GFP revealed that siGFP-liposome treated cells produced lower levels of protein in all silencing groups. Very interestingly, according to ‘pre- and co-silencing’ protocols, siNeg enhanced GFP expression compared to Ad alone as shown by both western blotting and fluorescence intensity data (p<0.05 versus Ad).

In order to assure simultaneous delivery of both siRNA and Ad, we attempted to co-envelope the virus and nucleic acid in a single vector, calling these as ‘dual-activity vectors’. Co-enveloped Ad and siRNA vectors were prepared using the lipid film hydration method followed by sonication. We have previously engineered and fully characterized enveloped Ad in different lipid bilayers [6]–[8]; and showed the importance of cationic and pH-sensitive nature for artificial envelopment. Here, we focused on the simultaneous envelopment of Ad and siRNA within DC-Chol:DOPE lipid bilayers. DC-Chol:DOPE lipid bilayers have been extensively studied as a cationic pH-sensitive gene delivery system, and showed promising results *in vitro* and *in vivo* [11]–[13].

**Figure 2:**
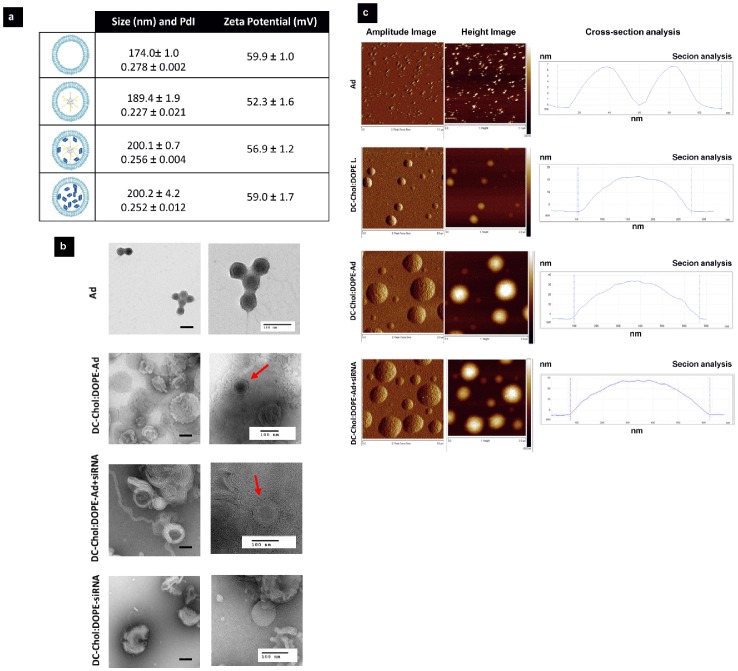
Characterization of co-enveloped Ad-siRNA vectors by DLS, TEM and AFM. The naked Ad, enveloped Ad, co-enveloped Ad-siRNA and enveloped siRNA are the vectors examined in this study. (a) The mean average diameter (nm), polydispersity index and surface charge (mV) for each vector were obtained by DLS. (b) Samples were analyzed by TEM imaging. Scale bars represent 100 nm. Red arrows indicate enveloped Ad vectors. (c) Samples were analyzed by AFM imaging.

DLS measurements showed moderate increase in hydrodynamic diameter, the enveloped Ad size increased from 189.4 nm to 200.1 nm following inclusion of siRNA in the vectors (Fig. 2a). Empty liposomes and siRNA encapsulated liposomes were 174.0 nm and 200.2 nm, respectively. Zeta potential measurements showed that all vectors had positively charged surface potential (Fig. 2a), in accordance with the characteristics of the lipid molecules used to form the bilayers.

The structural characteristics of dual-activity vectors were further elucidated by transmission electron microscopy (TEM) (Fig. 2b) and atomic force microscopy (AFM) (Fig. 2c). TEM imaging indicated the characteristic electron dense icosahedron capsid region for naked Ad. When DC-Chol:DOPE was used for envelopment, the virions appeared to be surrounded by concentric lipid bilayers. Similar structures were also obtained for co-enveloped Ad-siRNA vectors. Liposomes encapsulating only siRNA did not show vesicular structures similar to empty liposomes, which we attributed to the formation of lipoplexes between the positively charged lipid molecules and negatively charged siRNA. Consistent with our previous AFM imaging of DOTAP:Chol enveloped Ad [6], [8], the AFM imaging data offered experimental evidence of encapsulation of virions within liposomes, manifested by the significant height increases observed for DC-Chol:DOPE enveloped Ad compared to DC-Chol:DOPE liposomes alone or Ad virions alone. The interaction between Ad and AFM tip indicated that when tip retracted from Ad surface there were adhesive forces imposed on the tip (S1 Figure). Such forces are determined by the surface properties of Ad. However, we did not see such interactions when Ad or Ad+siRNA were enveloped by lipid bilayers. This further indicated that the Ad or Ad+siRNA were fully enveloped by a lipid bilayer. Moreover, co-envelopment of Ad and siRNA showed no significant further increase in overall vector dimensions corroborated by both TEM and AFM imaging.

**Figure 3:**
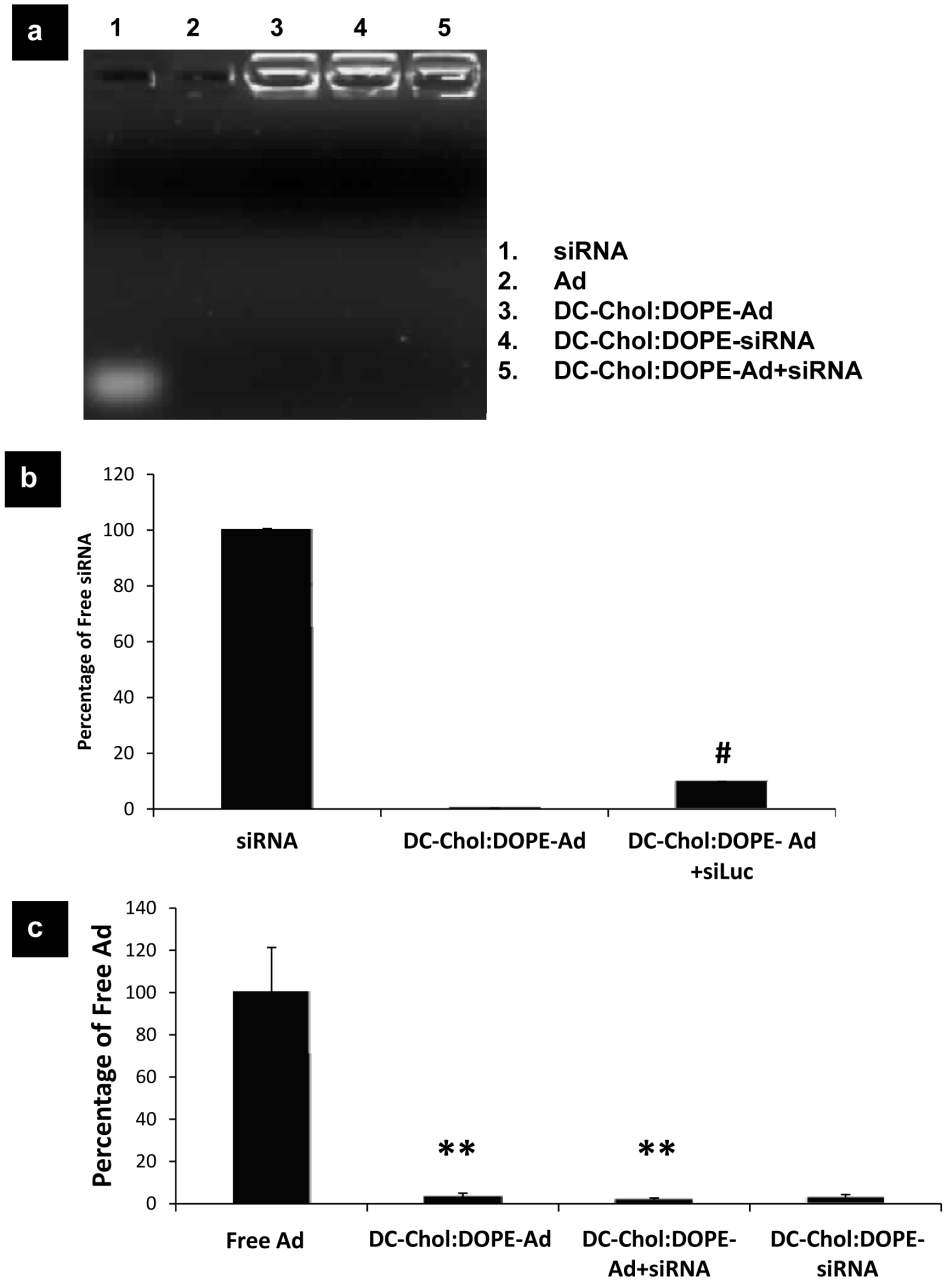
Envelopment efficiency of Ad and siRNA in dual-activity vectors. The naked Ad, free siRNA, DC-Chol:DOPE enveloped Ad, co-enveloped Ad-siRNA and enveloped siRNA were prepared. (a) Mobility shift assay was performed in order to detect the amount of free siRNA. (b) siRNA envelopment efficiency was quantified with Ribo green assay. (c) ELISA was used to quantify the percentage of free Ad. # p<0.05 versus free siRNA, **p<0.01 versus Ad.

Following characterization of these novel dual-activity vectors, we aimed to assess the envelopment efficiency of both Ad and siRNA. Mobility shift and Ribo green assays were used to detect the free siRNA after simultaneous envelopment of Ad and siRNA within lipid bilayers. The mobility shift assay was used to separate free or non-encapsulated siRNA from siRNA which is encapsulated within the liposome, and ethidium bromide was used to detect RNA molecules. Negatively charged siRNA migrates through the agarose gel and enveloped siRNA remains in the well, associated with the liposome. DC-Chol:DOPE liposomes have a net positive charge so would migrate towards the anode, however their size restricts entry into the agarose therefore liposomes and enveloped siRNA were retained in the well. Therefore the mobility shift assay in Fig. 3a indicated that when compared to free siRNA lane, dual-activity vectors successfully enveloped siRNA in DC-Chol:DOPE, even in the presence of Ad. Next, in order to quantify the envelopment efficiency, we used Ribo green assay (Fig. 3b). Free siRNA was used as a positive control whereas enveloped Ad was used as a negative control. When dual-activity vectors were incubated with Ribo green solution (before and after treatment with Triton X-100 in order to disrupt the lipid bilayer), only 10% of total siRNA included in the dual activity formulation was detected to be free. This result indicated that the envelopment efficiency was around 90% as determined by Ribo green assay (Fig. 3b). In order to determine the Ad envelopment efficiency, ELISA was carried out using anti-hexon antibodies to detect any unenveloped hexon regions on viral capsid (Fig. 3c). When Ad was enveloped, anti-hexon antibodies did not recognize the hexon regions of the virus particles. When siRNA was included, the efficiency did not change, and stayed above 90%. In conclusion, we achieved significantly high envelopment efficiency (above 90%) for both DC-Chol:DOPE-Ad and DC-Chol:DOPE-Ad+siRNA.

**Figure 4:**
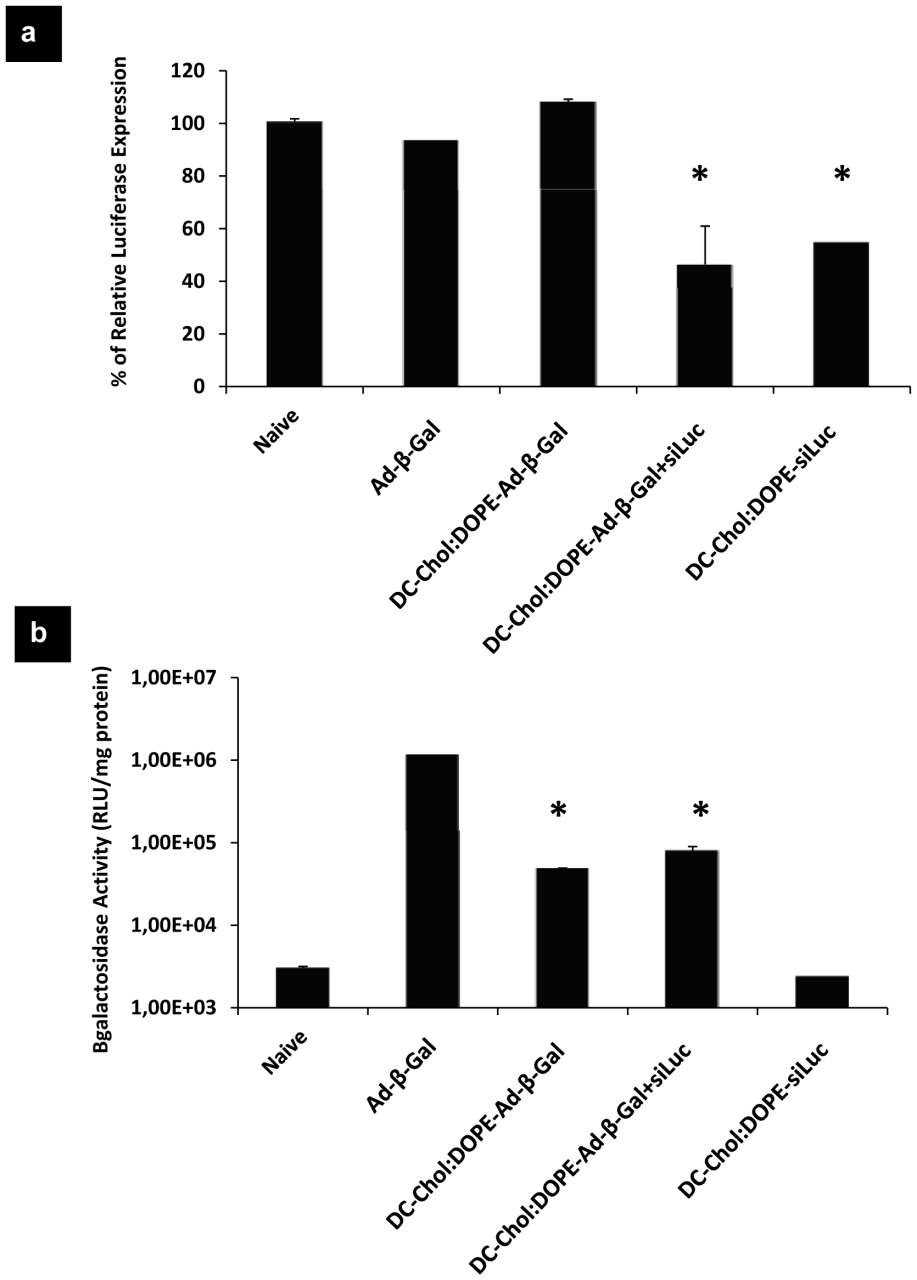
The biological activity of co-enveloped Ad and siRNA in DC-Chol:DOPE. The naked Ad, DC-Chol:DOPE enveloped Ad, co-enveloped Ad-siRNA and enveloped siRNA are the vectors examined in this study. A549-luc-A9 cells were transfected with these vectors and after 24 h, cells were lysed. (a) Luciferase assay was performed in order to measure the percentage of luciferase expression. (b) β-Gal expression was assessed by β-Gal assay. * p<0.05 versus Ad.

A luciferase expressing human lung carcinoma cell line (A549-luc-C8) was used to study the dual-activity vectors *in vitro*. This cell line is stably expressing luciferase gene, therefore we chose siLuc to silence luciferase and Ad-β-Gal to deliver β-Gal gene. Cells were incubated with the vectors for 3 h and 24 h after gene silencing was analyzed by luciferase assay. As seen in Fig. 4, percentage of relative luciferase activity was around 40–50% when cells were transfected by DC-Chol:DOPE-Ad+siRNA or DC-Chol:DOPE-siRNA. When the same cell lysates were analyzed for β-Gal activity, DC-Chol:DOPE envelopment showed a lower gene expression profile compared to naked Ad treated cells. Inclusion of siRNA in the dual-activity vector system did not alter the biological activity of enveloped Ad vectors, similar levels of β-Gal expression and luciferase silencing were obtained (Fig. 4). Negative control experiments were performed with a siNeg sequence, and the results indicated that the presence of siRNA in dual-activity vectors did not have any effect on the gene delivery capacity of these vectors (S2 Figure).

**Figure 5:**
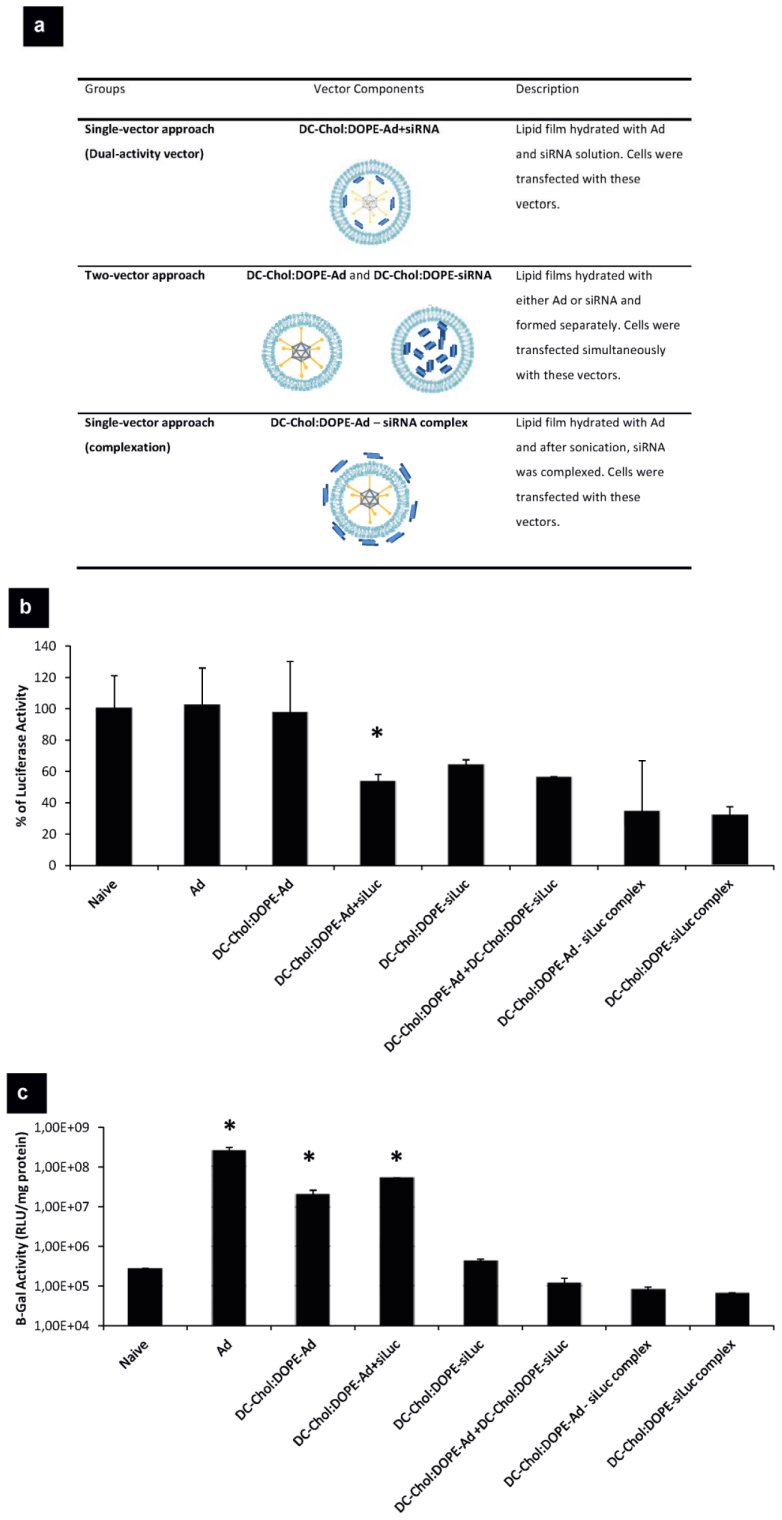
The comparison of dual-activity vectors with other gene delivery systems. (a) Dual-activity vectors were compared with different systems in terms of transgene expression and downregulation. (b) A549-luc-A9 cells were transfected with these vectors and after 24 h, cells were lysed. Luciferase assay was performed in order to measure the percentage of luciferase expression. (c) β-Gal expression was assessed by β-Gal assay. * p<0.05 versus naive.

These novel dual-activity vectors were prepared via lipid-film hydration method. DC-Chol:DOPE films were hydrated with siRNA and Ad solution simultaneously. We compared co-enveloped Ad-siRNA vectors with mixing, complexation and other controls as shown in Fig. 5a. When we treated cells with a two-vector approach (DC-Col:DOPE-Ad + DC-Chol:DOPE-siRNA vectors), same levels of luciferase silencing were obtained as with the dual-activity vector, however a very low β-Gal expression was seen using this two-vector approach (Fig. 5b,c). This result showed that delivery of genetic instructions with a two-vector approach could not be effective as dual-activity vectors. Rather than encapsulating siRNA together with Ad, siRNA was complexed to DC-Chol:DOPE-Ad vectors, in order to mimic our dual-activity vectors (single-vector approach by complexation). When A549-luc-C8 cells were treated with these vectors, luciferase gene silencing was variable among replicates and very low levels of β-Gal gene expression were obtained, unlike the novel dual-activity vectors (Fig. 5b,c). Overall, this data suggested that the dual-activity vectors are more effective for virus and siRNA delivery than other vectors tested in this study.

**Figure 6:**
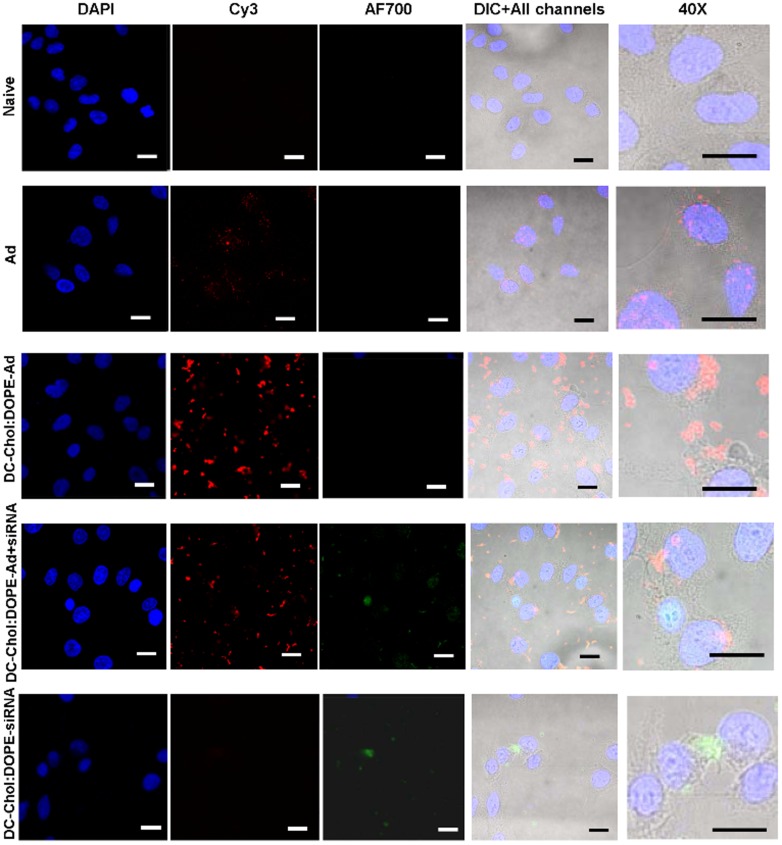
Cellular uptake profile of dual-activity vectors. Cy3-labelled Ad (1×10^10^ p/ml) and Alexa 700-labelled siNeg (1 nmol) were used in the envelopment process. Cells were incubated with Cy3-Ad, enveloped Cy3-Ad, co-enveloped Cy3-Ad and AF700-siNeg or enveloped AF700-siNeg. After 3 h in serum free media, cells were fixed and imaged under CLSM. Pseudo-DIC images (bottom right panels) were combined with a AF700 (bottom left panels), Cy3 (top right panels) and DAPI (top right panels) fluorescence images. Representative images were taken at 10x (scale bars  = 20 µm) and 40x (scale bars  = 5 µm).

Finally we studied the cellular uptake of dual-activity vectors in A549 cells. We treated the cells with different vectors for 3 h and imaged under CLSM (Fig. 6). Yellow channel was used to detect Ad which was labelled with Cy3 and far-red channel was used to track the siRNA-AF700 uptake in the cells. DAPI was used for nuclear staining. As expected, when cells were treated with naked Ad-Cy3, upon endosomal release, perinuclear co-localization was observed. However when cells were incubated with DC-Chol:DOPE-Ad-Cy3, the signals were enhanced possibly due to the aggregation resulted from cationic charges. Viral particles were detected mainly in the cytoplasmic space, some escaped from endosomes and migrated towards the perinuclear region. DC-Chol:DOPE-siRNA-AF700 treated cells showed green fluorescence from cytoplasmic region, as expected. When we co-enveloped Ad and siRNA within lipid bilayers, similar cellular uptake profile was obtained as single vectors. These results suggested that there was no interference between the genetic material transfer capacity and simultaneous envelopment of Ad and siRNA in DC-Chol:DOPE envelopes.

## Discussion

The basis of gene therapy is to alter a disease phenotype by introducing new genetic instructions into tissues and cells of patients to compensate for abnormal or missing functions. For several disorders, simultaneous gene transfer and knockdown by delivery of both genes and siRNA within the same cells may work synergistically to improve therapeutic outcomes. In this study, we attempted to illustrate the feasibility of combinatory adenoviral gene transfer with siRNA-mediated gene silencing by engineering dual-activity vectors.

Cancer gene therapy with simultaneous oncolytic adenovirus delivery or siRNA have recently gained popularity and there are a few pilot studies already combining these technologies [14]–[16]. *In vivo* studies have shown that such a dual- activity approach (oncolysis with silencing) can be considered promising for cancer therapy [1], [2], [17]. However, these studies engineer shRNA within oncolytic adenovirus genomes and achieve tumour therapy by intratumoural injections. The artificial adenovirus envelopment technology we have previously developed [6], [7] offers evasion of the immune system and provides a promising platform for systemic administration of re-targeted vectors [18]. We have previously reported that the HepG2 cellular uptake profile of enveloped Ad vectors is independent of blood coagulation factors *in vitro* [18]. *In vivo* studies proved that the envelopment can reduce hepatocyte transduction and thus improve hepatotoxicity profiles. Moreover, it has been shown that pH-sensitive artificial envelopes can allow triggerable release and endosomal escape of enveloped virions [8]. We aimed to widen the capabilities of this vector technology by the inclusion of siRNA nucleic acids to achieve concomitant gene silencing within the same cell transfected by the enveloped Ad virions.

The feasibility of gene delivery by Ad and the subsequent gene silencing by siRNA were first studied independently, using different vectors. We transfected cells with Ad-GFP and then attempted to knockdown the GFP transgene using liposome:siGFP complexes in three different treatment protocols as shown in Fig. 1. Fluorescence and western blot data of GFP showed that pre- and co-silencing exhibited more effective dual- activity compared to the post-silencing protocol. However, in the co-silencing protocol wider variation was obtained that could be explained by a possible interaction between Ad viral particles and liposome:siRNA complexes in the cell culture environment. This interaction may be responsible for the observed decrease in the levels of both GFP transfection and silencing activities within the cells. More importantly, combining Ad gene transfer with siRNA silencing in a two-vector approach as in our pre-, co- or post-silencing protocols is not practical and prone to irreproducibility, particularly in relation to clinical translation. To improve this, we developed a novel, dual activity vector by co-envelopment of Ad and siRNA within pH-sensitive DC-Chol:DOPE lipid bilayers.

Interestingly, treatment with siNeg showed higher levels of GFP expression compared to control groups. We hypothesize that the presence of siNeg during the transcription of the transgene can lead to overexpression of the protein product. This gene activation effect from siNeg sequences was also demonstrated in previous studies and has become a highly controversial topic in the RNAi research field. There are several hypothesis proposed for this unexpected activation effect [9], [10] such as the activation of noncoding regulatory regions in gene promoter, imbalance in bidirectional transcription levels or recruitment of Argonaute to the target gene promoter. Based on these observations, further systematic work on the role of siNeg in viral transgene activation is warranted.

Variety of materials is under exploration to address the challenges of *in vivo* delivery, including polymers, lipids, peptides, dendrimers and carbon nanotubes [19]–[21]. Formulation stability, safety and transfection efficiency should be considered carefully when designing these gene delivery vectors [22], [23]. In our dual-activity vectors, we co-enveloped Ad and siRNA within lipid bilayers in order to achieve efficient simultaneous gene transfection and silencing with improved vector stability. It is well known that encapsulating siRNAs rather than complexation with cationic liposomes is a more promising option to overcome obstacles such as low stability on serum, inefficient internalization by target cells and protection from nuclease activities [24], [25]. Furthermore, numerous studies have reported that incorporation of plasmid DNA into siRNA lipoplexes can form stable vector systems and enhance the silencing effect of specific genes [26]–[28]. These reports support the concept of combining Ad and siRNA within the same lipid bilayer. DLS and TEM/AFM microscopy showed that stable dual-activity nanoscale vectors with a mean diameter between 100–200 nm could be obtained. Another study reported that simultaneous gene transduction and silencing can be achieved by chimeric nanoparticles in which an adeno-associated viral particle was shielded with an acid-degradable, siRNA-encapsulating polyketal shell [29]. In this study, *in vitro* experiments suggested that co-envelopment of Ad and siRNA within lipid bilayers resulted in biologically functional dual-activity gene therapy vectors, compared to single vectors alone. We speculate that delivery of two different vectors to a single cell would happen at a lower frequency and with a higher variability than using a single vector to co-deliver different genetic material simultaneously. Alternatively, presence of different liposomal systems (one with Ad and the other with siRNA) could interfere with each other during cellular uptake and decrease efficiency. Another reason could be the possible off-target effect of siRNA on CMV promoter of Ad through NF-κB activation. Further studies are needed to explain this observation at the molecular level. In this study, β-gal expression levels of enveloped Ad vectors were lower than the naked virions, since the pH-sensitive cationic lipid bilayer (DC-Chol:DOPE) used in this study was not as effective as the previously reported pH-sensitive anionic envelope DOPE:CHEMS. Therefore future studies could further improve the transfection efficiency of these dual-activity vectors by changing the characteristics of artificial lipid bilayers.

Dual-activity vectors offer relatively facile development that does not require extensive genetic engineering as in the case of other double-targeting vector systems employing oncolytic Ad with shRNA within the viral genome. Variety of siRNA sequences can be co-enveloped enhancing the flexibility and multiplicity of gene targets for such vectors. Additionally, it has been observed that shRNA delivery via viral vectors is not effective in tumours with low concentration levels of Dicer which is required for the processing of shRNA [30]. Therefore, co-envelopment of Ad-siRNA vector bypasses the need for the Dicer, and offers an advantage to the other dual-activity vector systems to provide a new generation of multi-functional gene transfer platform.

## Materials and Methods

### Materials

Recombinant Adenovirus type 5 vectors (Ad-β-Gal and Ad-GFP) were purchased from the Baylor College of Medicine, Vector Development Laboratory in Texas, USA. Ad.β-Gal encodes β-Galactosidase and Ad-GFP carries enhanced green fluorescent protein reporter gene under the control of cytomegalovirus (CMV) promoter. Viral stocks were obtained at a concentration of 5×10^12^ particles/ml (5×10^12^ p/ml), which were equivalent to 1.2×10^11^ plaque-forming units (pfu)/ml, in 20 mM HEPES, 150 mM NaCl and 10% glycerol at pH 7.8 and stored at −80°C until ready for use. A549-luc-C8 cell line (Caliper Life Scienves, USA) is a luciferase expressing cell line derived from A549 by stable transfection of the North American Firefly Luciferase gene expressed from the CMV promoter. A549-luc-C8 were maintained in RPMI-1640 and human hepatocarcinoma HepG2 cells (ATCC, USA) were maintained in Minimum Essential Medium (MEM), both supplemented with 10% fetal bovine serum (FBS), 50 U/ml penicillin, 50 µg/ml streptomycin, 1% L-glutamine and 1% non-essential amino acids at 37°C in 5% CO_2_. DC-Cholesterol (DC-Chol, 3ß-[N-(N',N'-dimethylaminoethane)-carbamoyl]cholesterol hydrochloride) and DOPE (1,2-dioleoyl-*sn*-glycero-3-phosphoethanolamine) lipids were purchased from Avanti Polar Lipids (Alabama, USA). Bovine serum albumin (BSA) and HRP-labelled anti mouse IgG were obtained from Sigma, UK. BCA Protein assay kit 1-Step Ultra TMB-ELISA substrate and ECL detection system were purchased from Fisher, UK. Mouse anti-hexon antibody (ab8251) was from Abcam plc, UK.

### Co-envelopment of Ad and siRNA in lipid bilayers

Dual activity vectors were prepared with lipid film hydration method similar to artificial envelopment of Ad. In brief, Dc-Chol:DOPE (at 1∶3 molar ratio with 3 mM total lipid concentration) were dissolved in 4∶1 chloroform: methanol and lipid films were prepared using a rotovaporator (Buchi, Switzerland). Lipid films were hydrated with 1 ml of 5×10^9^ p/ml Ad- β-Gal and 0.625 nmol siLuc or siNeg in 20 mM HEPES, 150 mM NaCl buffer at pH 7.2. For TEM and AFM analysis, lipid films were hydrated with 1×10^10^ p/ml Ad- β-Gal and 0.625 nmol siLuc. For CLSM studies, Cy3-labelled Ad (1×10^10^ p/ml) and Alexa 700-labelled siNeg (1 nmol) were used. Following lipid film hydration, co-enveloped vectors were sonicated in cuphorn sonicator (Sonics, Switzerland) for 10 cycles of 4 min (15 sec pulse on and 5 sec pulse off) sonication at 20% amplitude. Samples were left in the fridge for annealing for 2 h.

### Nanoparticle sizing and zeta potential measurements by dynamic light scattering (DLS)

Size and zeta potential analysis of enveloped Ad, enveloped siRNA, empty liposomes and co-enveloped Ad-siRNA samples were conducted in Zetasizer Nano ZS (Malvern Instruments, UK). Size was measured in 1 mL cuvettes and samples were diluted with 20 mM HEPES, 150 mM NaCl buffer at pH 8.0. Zeta potential was measured in disposable zetasizer cuvettes, and samples were diluted in distilled water. Three measurements for sizing and five measurements for zeta potential were taken.

### Evaluation of Ad envelopment by ELISA

High-bond ELISA plates were overnight coated with 100 µl of Ad, enveloped Ad or dual-activity vector samples at different concentrations in carbonate/bicarbonate buffer, pH 9.0 at 4°C. Plates were blocked with 1.5% BSA in TBS for 2.5 h. Wells were washed 3 times with TBS. Mouse anti-hexon antibody at a concentration of 2.5 µg/ml was used as a detection antibody. After 2.5 h incubation at 37°C, wells are washed 3 times with TBS. 100 µl of HRP-linked anti-mouse IgG at 1∶10000 dilution was added to the wells. 1-Step Ultra TMB-ELISA was used as a detection system. Sulphuric acid (2 M) was added to stop reaction and absorbance was measured at 450 nm in Fluostar Omega plate reader (BMGLabtech,UK).

### Transmission electron microscopy (TEM)

Vectors were prepared as described above. Samples were concentrated with 30000 molecular weight cut off (MWCO) spin columns and dried onto a gold grid and stained with 1% aqueous uranyl acetate. Images were obtained using a CM120 BioTwin electron microscope (Philips, USA).

### Atomic force microscopy (AFM)

Vectors were prepared as described above. Samples were concentrated five times with 30000 molecular weight cut off (MWCO) spin columns and 20 µl of a suspension containing 10^11^ Ad particles/ml was deposited on the surface of freshly cleaved mica (Agar Scientific, Essex, UK), and viruses were allowed to adsorb for 15 min. Unbound viruses were removed by washing with filtered dH_2_O. Samples were then dried under a nitrogen stream. Imaging was carried out in TappingMode using a Multimode AFM, E-type scanner, Nanoscope IV controller, Nanoscope 5.12b control software (all from Veeco, Cambridge, UK), and a silicon tapping tip (NSG01, NTI-Europe, Apeldoorn, The Netherlands) of 10 nm curvature radius, mounted on a tapping-mode silicon cantilever with a typical resonant frequency of 150 kHz and a force constant of 5.5 N/m, to image 5×5 µm square areas of the mica surface with a resolution of 512×512 pixels and a scan rate of 1 Hz. All AFM images were performed in air.

### Mobility Shift Assay

Agarose gels (1%) were prepared in TBE buffer and ethidium bromide was added (0.5 µg/ml). Ad, free siRNA, enveloped Ad, enveloped siRNA, co-enveloped Ad-siRNA and empty liposome samples were analyzed by agarose gel electrophoresis system (Bio-Rad, UK). Bands on gels were visualized by UV illumination and imags captured using the Syngene Gel Box (Syngene, UK).

### Ribo green assay

Dual activity vectors were prepared as described above. In order to detect siRNA envelopment efficiency, 100 µl from each sample, was treated with 0.01% Triton X-100 and used in a high range assay (20 ng/mL to 1 µg/mL RNA) with the e Quant-iT RiboGreen RNA assay. Instructions supplied with the kit were followed and the sample fluorescence was measured using a fluorescence microplate reader (excitation ∼480 nm, emission ∼520 nm). Values were normalized with a blank and percentage of siRNA envelopment was determined.

### *In vitro* experiments

C33-a cells (human cervix carcinoma cell line) were seeded onto 24-well plates and incubated at 37°C, 5% CO2, overnight. Three different treatment groups to study the activity of two-vector approach were studied: “pre-silencing”, “co-silencing” and “post-silencing” as shown in Fig. 1a. In “pre-silencing group”, cells were first incubated with 100 nM siGFP or siNeg complexed with DOTAP:Chol (2∶1 mM) liposomes in serum free media and the next day, cells were transfected with Ad-GFP (10^8^ p/mL) for 3 h. In the “co-silencing group”, Ad-GFP and siRNA complexed with liposomes were added together to the wells and incubated for 3 h. In “post-silencing” group, cells were transfected with Ad-GFP on the first day and silenced with siGFP complexed with either DOTAP:Chol liposomes a day after. For the siNeg experiments, only “pre-silencing” treatment was followed. Gene expression was analyzed via β-Gal assay or western blotting.

For the dual-vector approach, A549-Luc-C8 cells (luciferase expressing human lung carcinoma cell line) were transfected with DC-Chol:DOPE (1∶3, 3 mM) co-enveloped Ad- β-Gal (5×10^9^ p/ml) and siLuc or siNeg (0.625 nmol). After 3 h, media was replaced with fresh complete media. Gene expression was analyzed via β-Gal assay and gene silencing was assessed by Luciferase Assay Kit.

### Confocal laser scanning microscopy imaging (CLSM) of GFP expression and cellular uptake studies

Reporter gene activity was assessed with Confocal Laser Scanning Microscopy (CLSM) for the cells transfected with Ad-GFP. 24 h after transfection, plates were observed under confocal microscopy, Zeiss LSM 510 Meta laser scanning confocal microscope, equipped with a 30 mW argon laser, a 1 mW, 543 nm HeNe laser, and a 5 mW, 633 nm HeNe laser. Samples were analyzed using two channel confocal laser scanning microscopy to obtain a pseudo-DIC image combined with a GFP fluorescence image. Representative images were taken at 10x and 40x.

Cellular uptake of co-enveloped Ad siRNA was studied in A549 cells. Cells were seeded onto glass coverslips. Next day, Cy3-labelled Ad (1×10^10^ p/ml) and Alexa 700-labelled siNeg (1 nmol) were used in the envelopment process. Cells were incubated with Cy3-Ad, enveloped Cy3-Ad, co-enveloped Cy3-Ad and AF700-siNeg or enveloped AF700-siNeg. After 3 h in serum free media, cells were washed 3 times with PBS and fixed with cold methanol. Coverslips were mounted on glass slides and analyzed using four channel confocal laser scanning microscopy to obtain a pseudo-DIC image combined with fluorescence signals. Representative images were taken at 10x and 40x.

### Analysis of gene silencing by western blotting

Cells lysates were used to determine GFP protein levels by Western blotting. Total protein concentration was assessed with BCA Protein assay kit. 100 µg protein was taken from each supernatant and resolved on 12% SDS- polyacrylamide gel electrophoresis (PAGE) and transferred to Hybond ECL nitrocellulose membranes. After blocking in 5% non-fat dry milk overnight, the blots were incubated with rabbit polyclonal GFP antibody in 1∶5000 dilution. HRP-linked anti-rabbit IgG at 1∶1000 dilution was used as secondary antibody. The specific bands were detected with ECL detection system at Syngene Gel Box (Synege,UK).

### Statistical Analysis

In vitro experiments were performed in triplicates on at least three independent occasions. In vivo experiments were performed with at least four animals per group. Statistical analysis was performed by analysis of variance and Tukey's pairwise comparison using SPSS software, version 16.0.

## Supporting Information

S1 Figure **Deformation analysis of co-enveloped Ad-siRNA vectors by AFM.** The naked Ad, enveloped Ad, co-enveloped Ad-siRNA and enveloped siRNA are the vectors examined in this study. Samples were analyzed by AFM imaging.
(PDF)

S2 Figure **The effect of siNeg on the gene delivery capacity of dual-activity vectors.** A scrambled siRNA sequence was used in the dual activity vectors. A549-luc-A9 cells were transfected and after 24 h, cells were lysed. Luciferase assay was performed in order to measure the (a) luciferase activity and (b) percentage of luciferase expression.
(PDF)
