# Supplementary figures and images for: Development of Dual-Activity Vectors by Co-Envelopment of Adenovirus and SiRNA in Artificial Lipid Bilayers
Source: PLoS One. 2014 Dec 12;9(12):e114985. doi: 10.1371/journal.pone.0114985 (PMC4264847; doi:10.1371/journal.pone.0114985)

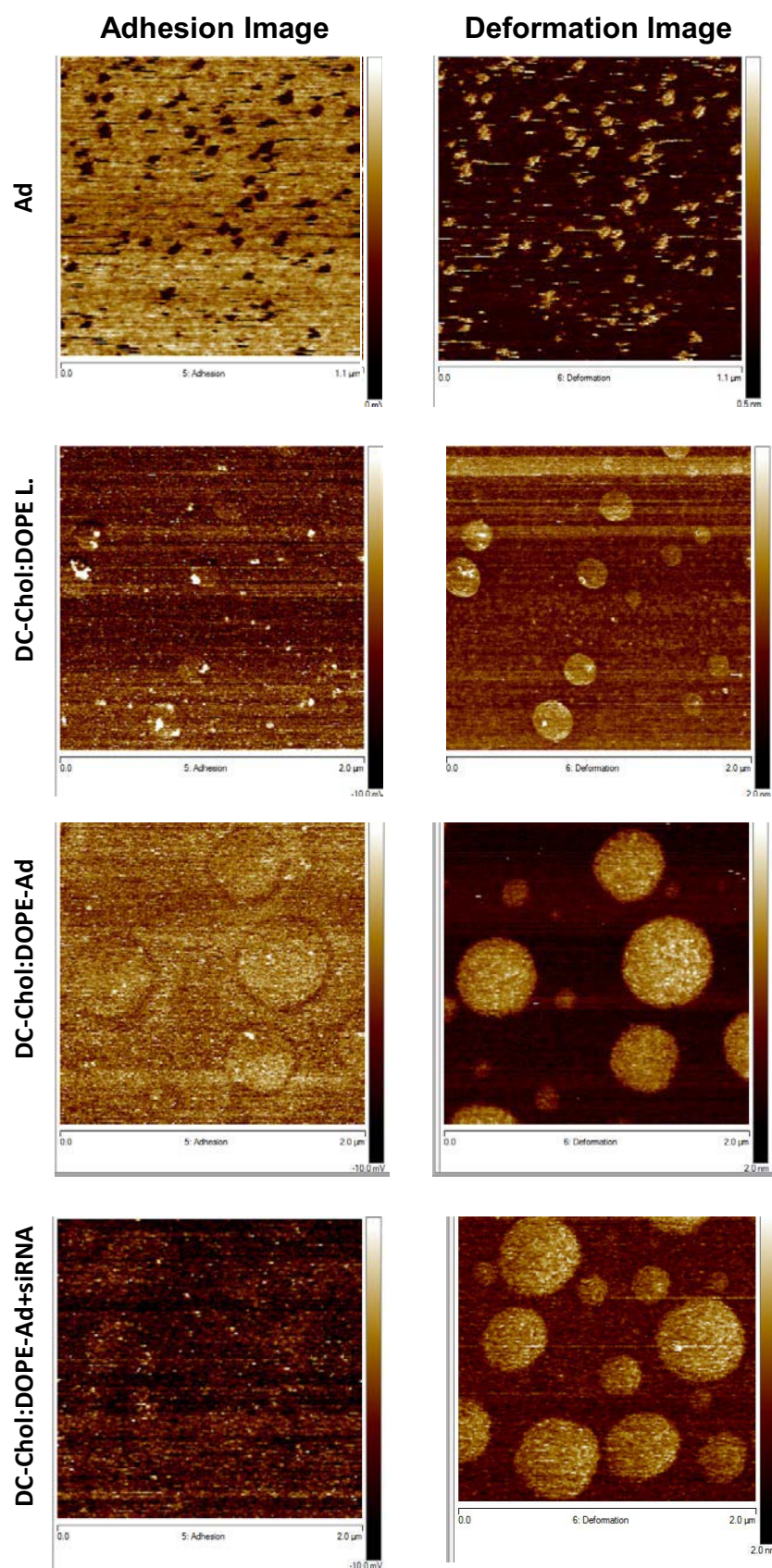

**Figure S1: Deformation analysis of co-enveloped Ad-siRNA vectors by TEM**

Supplement: S1 Figure — Deformation analysis of co-enveloped Ad-siRNA vectors by AFM. The naked Ad, enveloped Ad, co-enveloped Ad-siRNA and enveloped siRNA are the vectors examined in this study. Samples were analyzed by AFM imaging. (PDF) [file pone.0114985.s001.pdf]

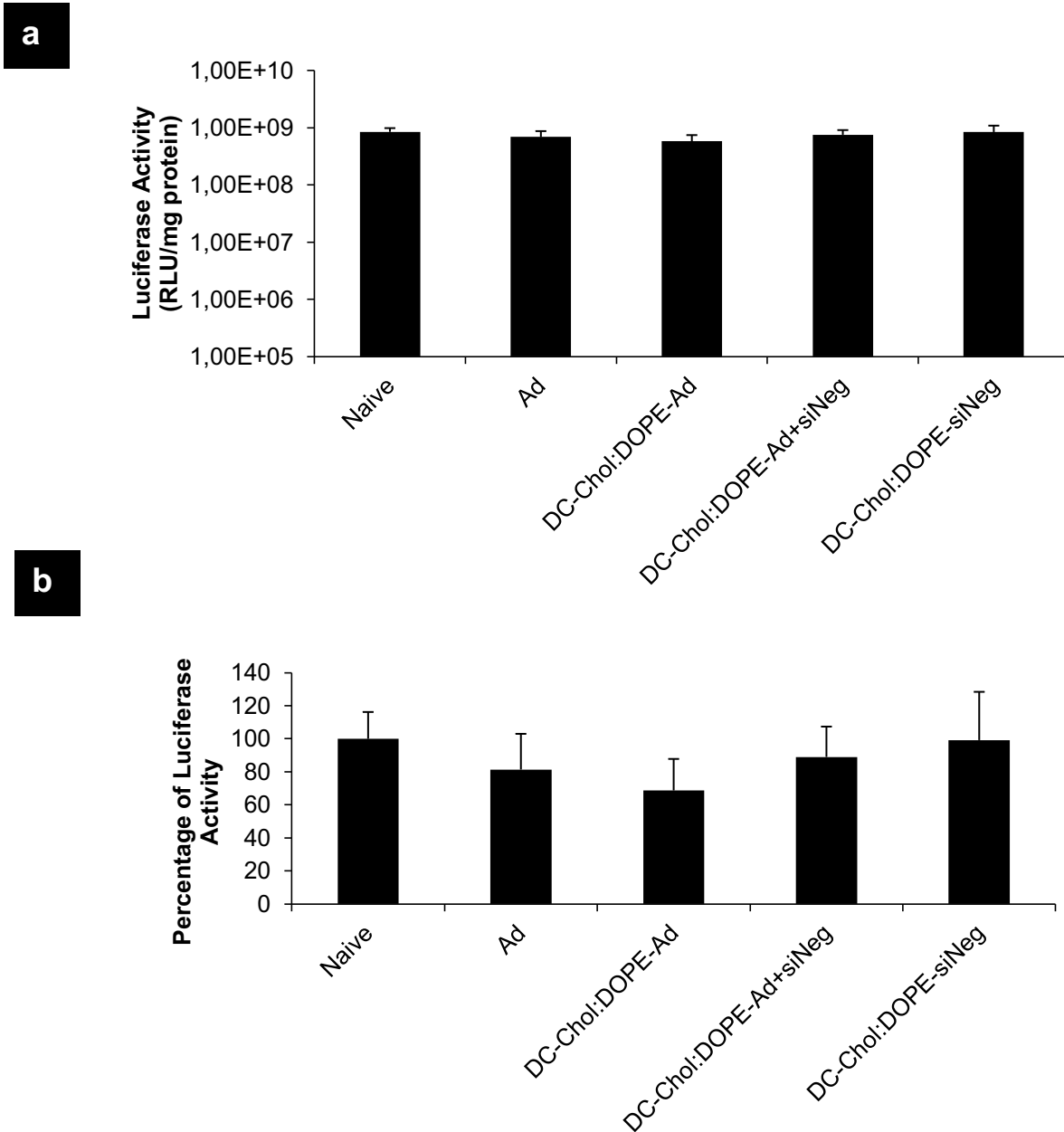

**Figure S2: The effect of siNeg on the gene delivery capacity of dual-activity vectors.**

Supplement: S2 Figure — The effect of siNeg on the gene delivery capacity of dual-activity vectors. A scrambled siRNA sequence was used in the dual activity vectors. A549-luc-A9 cells were transfected and after 24 h, cells were lysed. Luciferase assay was performed in order to measure the (a) luciferase activity and (b) percentage of luciferase expression. (PDF) [file pone.0114985.s002.pdf]
